# Regulatory T cells, damage-associated molecular patterns, and myeloid-derived suppressor cells in bronchoalveolar lavage fluid interlinked with chronic obstructive pulmonary disease severity: An observational study

**DOI:** 10.1097/MD.0000000000029208

**Published:** 2022-06-10

**Authors:** Beata Brajer-Luftmann, Mariusz Kaczmarek, Agata Nowicka, Marta Stelmach-Mardas, Magdalena Wyrzykiewicz, Senan Yasar, Tomasz Piorunek, Jan Sikora, Halina Batura-Gabryel

**Affiliations:** a Department of Pulmonology, Allergology and Pulmonary Oncology, Poznan University of Medical Sciences, Szamarzewskiego 84 Street, Poznan, Poland; b Department of Cancer Immunology, Poznan University of Medical Sciences, Garbary 15 Street, Poznan, Poland; c Gene Therapy Laboratory, Department of Cancer Diagnostics and Immunology, Greater Poland Cancer Centre, Garbary 15 Street, Poznan, Poland; d Department of Treatment of Obesity, Metabolic Disorders and Clinical Dietetics, Poznan University of Medical Sciences, Szamarzewskiego 84 Street, Poznan, Poland; e Department of Clinical Immunology, Poznan University of Medical Sciences, Rokietnicka 5D Street, Poznan, Poland; f The Christ Hospital Heart and Vascular Center, The Carl and Edyth Lindner Center for Research and Education, Cincinnati, OH.

**Keywords:** BALF, COPD, galectin, HSP27, MDSC, PFT, Treg

## Abstract

The role of regulatory T cells (Tregs), damage-associated molecular patterns (DAMPs), and myeloid-derived suppressor cells (MDSCs) in the mechanism of innate and adaptive immune responses in chronic obstructive pulmonary disease (COPD) is not well understood.

Evaluating the presence of Tregs in the bronchoalveolar lavage fluid (BALF) and peripheral blood in patients with COPD, and assessment of the relationship between Tregs, MDSCs, and DAMPs as factors activating innate and adaptive immune responses. Description of the association between immune and clinical parameters in COPD.

Thirty-one patients with COPD were enrolled. Clinical parameters (forced expiratory volume in one second [FEV1], forced vital capacity, total lung capacity [TLC], diffusion capacity of carbon monoxide, and B-BMI, O-obstruction, D-dyspnea, E-exercise [BODE]) were assessed. Tregs and MDSCs were investigated in the BALF and blood using monoclonal antibodies directly conjugated with fluorochromes in flow cytometry. The levels of defensin (DEF2), galectin-1 (Gal-1), galectin-3 (Gal-3), galectin-9 (Gal-9), heat shock protein-27 (HSP27), and surfactant protein A were assessed via sandwich enzyme-linked immunosorbent assay.

The percentage of Tregs was significantly higher in the blood than in the BALF, in contrast to the mean fluorescence intensity of forkhead box P3 (FoxP3). Significant associations were observed between Tregs and HSP27 (r = 0.39), Gal-1 (r = 0.55), Gal-9 (r = −0.46), and MDSCs (r = −0.50), and between FoxP3 and Gal-1 (r = −0.42), Gal-3 (r = −0.39), and MDSCs (r = −0.43). Tregs and clinical parameters, including FEV1%pred (r = 0.39), residual volume (RV)%pred (r = −0.56), TLC%pred (r = −0.55), RV/TLC (r = −0.50), arterial oxygen saturation (r = −0.38), and arterial oxygen pressure (r = −0.39) were significantly correlated. FoxP3 was significantly interlinked with RV/TLC (r = −0.52), arterial oxygen pressure (r = 0.42), and BODE index (r = −0.57).

The interaction between innate and adaptive immune responses in patients with COPD was confirmed. The expression of Tregs in BALF may have prognostic value in patients with COPD. The conversion of immune responses to clinical parameters appears to be associated with disease severity.

## 1 Introduction

The inflammation that occurs in chronic obstructive pulmonary disease (COPD) appears to be a modification of the normal inflammatory response to chronic irritants. The mechanism for this amplified inflammation is not yet understood and can be modified by genetic predisposition.^[1]^ COPD may develop both in smoker and non-smoker groups^[1]^ and persist even when smoking is stopped, suggesting that there are self-perpetuating mechanisms, although these have not yet been elucidated.^[2]^

The nature of the inflammatory response in COPD has not yet been clearly explained, but it probably involves both innate and adaptive immune responses.^[3]^ Cigarette smoke and other irritants inhaled into the airways may activate surface macrophages and airway epithelial cells to release multiple chemotactic mediators, particularly chemokines, which attract circulating neutrophils, monocytes, and lymphocytes into the lungs.^[3]^ One hypothesis suggests that cigarette smoke-induced airway epithelial immunogenic cell death is followed by damage-associated molecular pattern (DAMP) release and subsequent triggering of the innate and adaptive immune responses in COPD.^[4]^ The characteristic pattern of inflammation in COPD patients is associated with increased numbers of neutrophils, macrophages, T lymphocytes (predominantly TC1, T helper 1 [TH1], and T helper 17 [TH17] cells), and B lymphocytes in the airway lumen.^[3]^ Regulatory T cells (Tregs) in adaptive immunity in COPD and their association with spirometry parameters have already been investigated.^[5–10]^ Furthermore, some other cell types in innate immunity are also involved in COPD pathogenesis, such as dendritic cells and myeloid-derived suppressor cells (MDSCs).^[11,12]^ The connection between adaptive and innate inflammatory immune responses, mediated by different immunological cells and their mediators, may play a pivotal role in the development and progression of COPD.^[13,14]^

Therefore, the present study investigated the presence of Tregs in the bronchoalveolar lavage fluid (BALF) and peripheral blood (PB) of patients with COPD, evaluated the relationship between them, MDSCs, and DAMPs as factors activating innate and adaptive immune responses, and described an association between immune and clinical parameters in COPD.

## 2 Materials and methods

### 2.1 Study design

The study design was an observational, cross-sectional study conducted in accordance with the Helsinki Declaration. The study protocol was approved by the Bioethical Committee of Poznan University of Medical Science (No. 741/10). Written informed consent was obtained from all participants included in the study.

Patients were recruited from 2014 to 2015 at the Department of Pulmonology, Allergology and Pulmonary Oncology, Poznan University of Medical Sciences (Poland). In the whole COPD group, the anthropometrical parameters included assessing body weight and body height with an approximation of 0.1 kg and 0.5 cm (Seca digital scale 763; Seca, Hamburg, Germany). These parameters were used to calculate body mass index (BMI).^[15]^

Pulmonary function tests (PFTs), 6-minute walking test (6MWT),^[16]^ and dyspnea using the Modified Medical Research Council scale^[17]^ were performed. The B-BMI, O-obstruction, D-dyspnea, E-exercise (BODE)-index as a prognostic marker in COPD mortality prediction was calculated based on BMI, forced expiratory volume in one second (FEV1), 6MWT, and Modified Medical Research Council.^[18]^ Additionally, an arterial blood gas analysis was performed. Blood samples (1.5 mL to the self-filling syringe containing 60 IU dry heparin) were obtained from the patient's radial artery after 15 minutes resting in the sitting position (Siemens RAPIDLab 1265, Camberley, United Kindom 2012).^[19]^

All recruited patients underwent routine videobronchofiberoscopy (VBF) for diagnostic reasons in the bronchoscopy laboratory, being the university unit. All procedures were performed in accordance with good laboratory and diagnostic practices.

### 2.2 Study population

Thirty-one COPD patients in the stable disease were included in the study according to the following inclusion criteria: age >40 years, COPD diagnosed according to Global Initiative for Chronic Obstructive Lung Disease (GOLD) criteria^[20]^ with a relevant history confirmed with a postbronchodilator (FEV1/forced vital capacity [FVC] ratio <0.7) and current and former smokers. Before enrollment in the study, each patient was verified for exacerbation during last 6 weeks in accordance to the GOLD guidelines.^[20]^

The indication for using the VBF in the studied group of patients was microbiological evaluation.^[21]^ The exclusion criteria were as follows: other pulmonary disorders such as asthma, tuberculosis, pulmonary thromboembolism, interstitial pulmonary lesions, cancer diseases, autoimmune diseases, COPD exacerbation during last 6 weeks, active infection, and contraindications to the use of VBF.

### 2.3 Methods

#### 2.3.1 Pulmonary function tests

The spirometry, body plethysmography, and the diffusion capacity of carbon monoxide (DLCO) were performed using Master Screen Body/Diffusion Jaeger device (Erich Jaeger GmbH, Würzburg, Germany) by an experienced technician according to the current American Thoracic Society/European Respiratory Society guidelines.^[22]^ Spirometry was performed 15 to 30 minutes after the inhalation of a short-acting bronchodilator. PFT results were presented as % predicted according to the Global Lung Function Initiative references.^[23]^ Airflow limitation was defined as a postbronchodilator FEV1/FVC ratio <0.70.^[20]^

#### 2.3.2 VBF and BALF

VBF was performed by an experienced pulmonologist/bronchoscopist using a flexible video bronchoscope (BF-H190 Olympus, Tokyo, Japan). The procedures were performed according to the British Thoracic Society guidelines, which included information on indications and contraindications.^[21,24,25]^ Finally, BALF samples were collected according to international guidelines.^[26–28]^ The VBF was wedged in the segmental or subsegmental bronchus of the middle lobe. The bronchus was lavaged with 50 mL aliquots of sterile saline solution at 37°C, and then the fluid was aspirated. Furthermore, two 50 mL aliquots of saline solution at 37°C were instilled and aspirated in the same way.^[26–28]^

#### 2.3.3 Blood samples

PB samples were collected in tubes containing ethylenediaminetetraacetic acid (9 mL) for cytometric immunophenotyping. Blood samples for soluble analyte assessment were collected in a tube without anticoagulant (9 mL) and centrifuged at 2500 rpm for 10 minutes at 4°C. The obtained serum samples were immediately frozen at −70°C for subsequent investigations.^[12,29]^

#### 2.3.4 Immunophenotypic assessment

**Figure 1:**
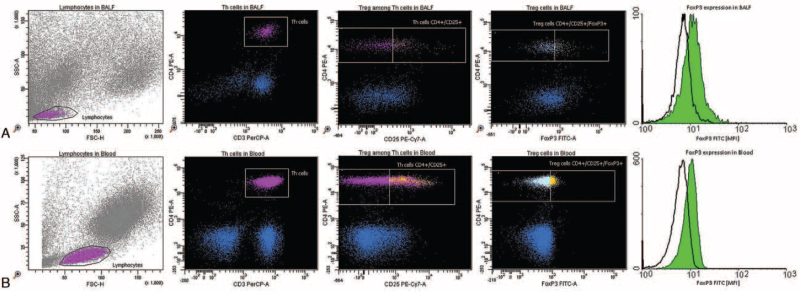
Tregs assessment according to the immunophenotype in (A) bronchoalveolar lavage fluid (BALF); (B) peripheral blood samples. The Tregs immunophenotypic pattern was defined as CD3^+^/CD4^+^/CD25^high^/FoxP3^+^. Tregs = regulatory T cells.

Fresh unfixed cells from BALF were immunophenotyped using a flow cytometer. The estimation of antigenic determinants, which are characteristic of Tregs and MDSC populations, was performed using monoclonal antibodies directly conjugated with fluorochromes (Fig. 1).

Tregs were assessed according to the immunophenotype: CD3^+^/CD4^+^/CD25^high^/FoxP3^+^,^[30]^ while MDSCs were defined as cells with SSC^low^/Lin-1^neg^/HLA-DR^neg/low^/CD11^+^/CD33^+^/CD45^+^.^[12,31]^ Cytometric analysis was performed using appropriate isotype controls. Samples for cytofluorometric analyses were prepared as follows: initially, BALF samples were filtered to remove any mucus, blood clots, cell aggregates, and tissue fragments. The cells were then centrifuged for 10 minutes at 1800 rpm to obtain pellets. Supernatants were removed, frozen, and stored for later evaluation of DAMP concentration. Antibodies against antigen characteristics of the tested cells were added to the cell pellets obtained from the BALF. Five microliters of each antibody per 2 × 10^5^ to 1 × 10^6^ cells were added to each tube. Samples without antibodies were used as negative controls. The antibodies used in the tests are grouped in Table 1. Cells mixed with antibodies were incubated for 15 minutes in the dark. Erythrocyte residues were lysed using 2 mL of hypotonic lysing solution (BD Biosciences). The addition of phosphate-buffered saline solution after 10 minutes changed the osmotic conditions and inhibited lysis. Finally, all lysed residues, morphotic particles, and soluble proteins were washed out by double centrifugation for 5 minutes at 1500 rpm. The expression of the transcription factor forkhead box P3 (FoxP3) was assessed by intracytoplasmic staining. Perm/Wash buffer (BD Biosciences) was used to permeabilize the cell membranes of the tested lymphocytes. Appropriately stained cells were acquired using a FACS Canto flow cytometer (BD Biosciences, San Jose, CA), and up to 50,000 events of each sample were collected and analyzed using FACS Diva software (BD Biosciences, San Jose, CA). The percentage of positive cells was also assessed.^[12,31]^ The total number of counted events in a single sample was 50,000.

**Table 1:** Specification of antibodies used in flow cytometry.

Antibody	Clone	Fluorochrome	Source
anti-Human Lineage Cocktail 1 (Lin-1) (cocktail of antibodies against)		FITC	BD Biosciences
CD3	SK7		
CD14	MfP9		
CD16	3G8		
CD19	SJ25C1		
CD20	L27		
CD56	NCAM16.2		
anti-HLA-DR	L243	PerCP	BD Biosciences
anti-CD11b	ICRF44	APC	BD Biosciences
anti-CD33	P67.6	PE-Cy7	BD Biosciences
anti-CD45	2D1	APC-Cy7	BD Biosciences
anti-CD3	SK7	PerCP	BD Biosciences
anti-CD4	SK3	PE	BD Biosciences
anti-CD25	2A3	PE-Cy7	BD Biosciences
anti-FoxP3	259D/C7	AlexaFluor488	BD Biosciences

#### 2.3.5 DAMPs concentration assessment

Serum samples and BALF supernatants were collected before analysis and stored at 80°C. All the studied DAMP molecules were evaluated in the BALF supernatant and serum samples. Frozen samples were thawed directly before concentration assessment. Concentrations of defensin (DEF2) (pg/mL), galectin-1 (Gal-1) (pg/mL), galectin-3 (Gal-3) (pg/mL), galectin-9 (Gal-9) (pg/mL), and heat shock protein-27 (HSP27) (ng/mL) were estimated using commercially available kits produced by USCN (Wuhan, China). Surfactant protein A (SP-A) (ng/mL) was assessed using the BioVendor kit (Brno, Czech Republic). All enzyme-linked immunosorbent assay tests were performed according to the manufacturer's instructions. BALF samples for the evaluation of SP-A were diluted to 1:100. DAMP concentrations were measured according to calibration curves. Reactions were stopped with H_2_SO_4_, and the absorbance of the colored products was measured at 450 nm using a Multiscan Bichromatic (Labsystems) microtitration reader. There was no standard range for these molecules, and the amount of BALF collected was variable. Therefore, using the Bradford test, the DAMP concentration values were calculated in comparison to the total protein (TP) concentration in the tested samples.^[31,32]^

### 2.4 Statistical analysis

The data were expressed as mean and standard deviation for normal distribution and as median and quartile in non-normal distribution. The normality of the distribution was checked using the Shapiro-Wilk test. The Wilcoxon test was used to analyze the differences between the assessed variables. The Spearman correlation coefficient was used to assess the presence of a relationship between % Tregs, % of Tregs among Th cells, FoxP3 expression, clinical parameters, DAMP concentration, and MDSCs. Statistically significant differences were defined as *P* < .05. All calculations were performed using Statistica 10 software (TIBICO Software Inc., Palo Alto, CA).

## 3 Results

### 3.1 Subject characteristics

Thirty-one patients with COPD were enrolled in the study. All patients were current or former smokers. The majority of patients who participated in the study belonged to GOLD 2010 stages II and III and presented a BODE index with a median of 3.0 points. Detailed characteristics of the study population are shown in Table 2.

**Table 2:** Characteristics of the study population (n = 31).

Analyzed variable (n = 31)	Median	Lower quartile	Upper quartile	Interquartile range
Sex (% of men)	80			
Age (yrs)	67.5	60.0	72.0	12.0
FEV1 (%pred)	49.5	34.0	62.3	28.3
FVC (%pred)	67.7	56.0	86.8	30.8
RV (%pred)	200.1	155.0	251.7	96.7
TLC (%pred)	117.4	105.7	139.3	33.6
RV/TLC (%pred)	66.9	57.6	71.0	13.4
DLCO (%pred)	37.5	24.9	59.4	34.5
SaO_2_ (%)	94.1	92.1	95.7	3.6
PaO_2_ (mm Hg)	66.9	61.1	74.1	13.0
BODE (0–10)	3.0	2.0	6.0	4.0
6MWT (m)	360.0	190.0	420.0	230.0
Smoking (pack years)	30.0	20.0	45.0	25.0

6MWT = 6-minute walking test, BODE = B-BMI, O-obstruction, D-dyspnea, E-exercise, DLCO = diffusion capacity of carbon monoxide, FEV1 = forced expiratory volume in one second, FVC = forced vital capacity, PaO_2_ = arterial oxygen pressure, RV = residual volume, SaO_2_ = arterial oxygen saturation, TLC= total lung capacity.

### 3.2 Tregs and Tregs FoxP3 frequencies in BALF and blood samples

**Figure 2:**
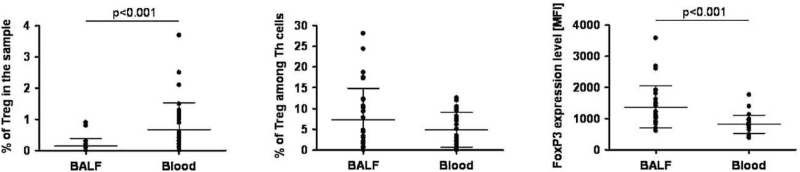
Tregs and Tregs FoxP3 frequencies in BALF and blood samples. BALF = bronchoalveolar lavage fluid, FoxP3 = forkhead box P3, Tregs = regulatory T cells.

Flow cytometric analysis showed that the percentage of Tregs was significantly higher in the blood than in the BALF of COPD patients (0.7% ± 0.9% vs 0.1% ± 0.3%, *P* < .001). The percentage of Tregs among Th cells was higher, but not significantly, in BALF compared with the blood sample (7.6% ± 15.5% vs 7.3% ± 7.5%, *P* < .001). Considerable differences in the FoxP3 expression levels (mean fluorescence intensity [MFI]) by the Tregs of different materials were observed. The MFI of FoxP3 in Tregs was significantly higher in BALF than in blood (1378.0% ± 670.4% vs 819.6% ± 292.7%, *P* < .001) (Fig. 2).

### 3.3 Soluble mediator concentrations

Defensine-beta, HSP27, SP-A, Gal-1, Gal-3, and Gal-9 concentrations and concentrations corrected by TP were detected in the BALF and serum. The results are shown in Table 3.

**Table 3:** Soluble mediators concentrations and concentration corrected by total protein in bronchoalveolar lavage fluid and serum.

Analyzed mediators	BALF	Serum	*P* value
Gal-1 (pg/mL)	50.0 ± 29.4	1171.8 ± 6286.6	<.001
Gal-1/TP (pg/mL)	86.8 ± 63.2	0.02 ± 0.02	<.001
Gal-3 (pg/mL)	0.3 ± 0.4	4.6 ± 4.5	<.001
Gal-3/TP (pg/mL)	0.5 ± 0.6	0.02 ± 0.01	<.001
Gal-9 (pg/mL)	0.9 ± 1.5	7.8 ± 7.5	<.001
Gal-9/TP (pg/mL)	1.2 ± 2.2	0.04 ± 0.03	.0251
BAL HSP27 (ng/mL)	1.4 ± 2.8	0.4 ± 0.6	.0140
BAL HSP27/TP (ng/mL)	2.5 ± 5.4	0.01 ± 0.02	<.001
BAL SP-A (ng/mL)	1.6 ± 3.1	0.02 ± 0.01	<.001
BAL SP-A/TP (ng/mL)	2.7 ± 6.5	0.02 ± 0.01	.4852
BAL DEF (pg/mL)	39.2 ± 20.6	589.2 ± 597.0	<.001
BAL DEF/TP (pg/mL)	65.0 ± 38.5	2.4 ± 2.3	<.001

Data are presented as mean ± standard deviation. BALF = bronchoalveolar lavage fluid, DEF = defensine-beta, Gal-1 = galectin-1, Gal-3 = galectin-3, Gal-9 = galectin-9, HSP27 = heat shock protein-27, SP-A = surfactant protein A, TP = total protein.

### 3.4 The percentage of MDSCs

This study revealed a significant difference between the percentage of MDSCs in all leukocytes and mononuclear cells (MCs) in BALF and PB. The percentage of MDSCs in BALF was lower than that in PB. The percentage of MDSCs among MCs assessed in BALF was also lower than that in PB (Table 4).

**Table 4:** The percentage of myeloid-derived suppressor cells among all leukocytes and mononuclear cells in bronchoalveolar lavage fluid and peripheral blood.

	BALF	Peripheral blood	*P* value
MDSCs (%)	0.7 ± 0.7	4.3 ± 3.7	<.001
MDSCs (% of MC)	4.7 ± 4.6	12.7 ± 3.5	<.001

Data are presented as mean ± standard deviation. BALF = bronchoalveolar lavage fluid, MC = mononuclear cell, MDSCs = myeloid-derived suppressor cells.

### 3.5 Relationship between the frequency of Tregs, DAMPs, and MDSCs

**Figure 3:**
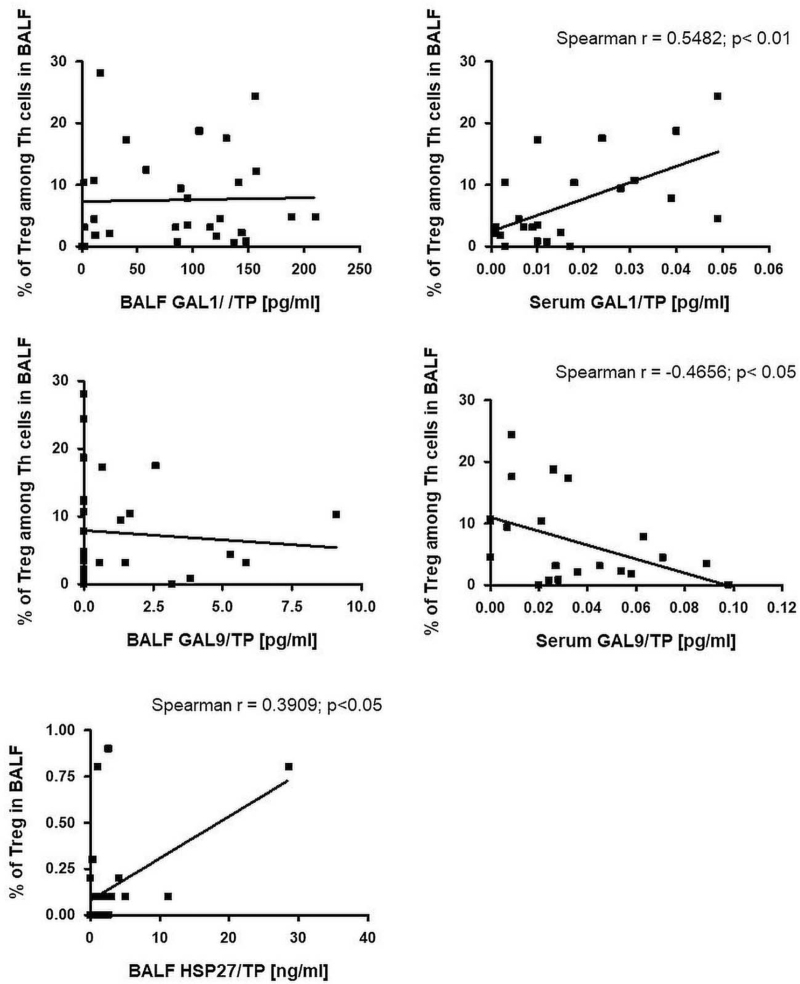
The correlation between Tregs in BALF and HSP27, Gal-1 and Gal-9 in BALF and serum. BALF = bronchoalveolar lavage fluid, Gal-1 = galectin-1, Gal-9 = galectin-9, HSP27 = heat shock protein-27, Tregs = regulatory T cells.

**Figure 4:**
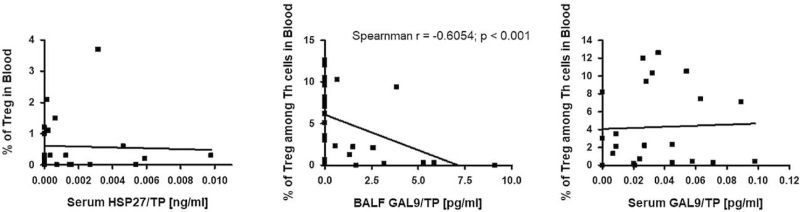
The correlation between Tregs in blood and HSP27 and Gal-9 in BALF and serum. BALF = bronchoalveolar lavage fluid, Gal-9 = galectin-9, HSP27 = heat shock protein-27, Tregs = regulatory T cells.

Statistical analysis showed a significant positive correlation between the percentage of Tregs in BALF and HSP27/TP concentration in BALF (r = 0.3909; *P* < .05) and a positive trend between the percentage of Tregs in blood and HSP27/TP concentration in serum (Figs. 3 and 4).

**Figure 5:**
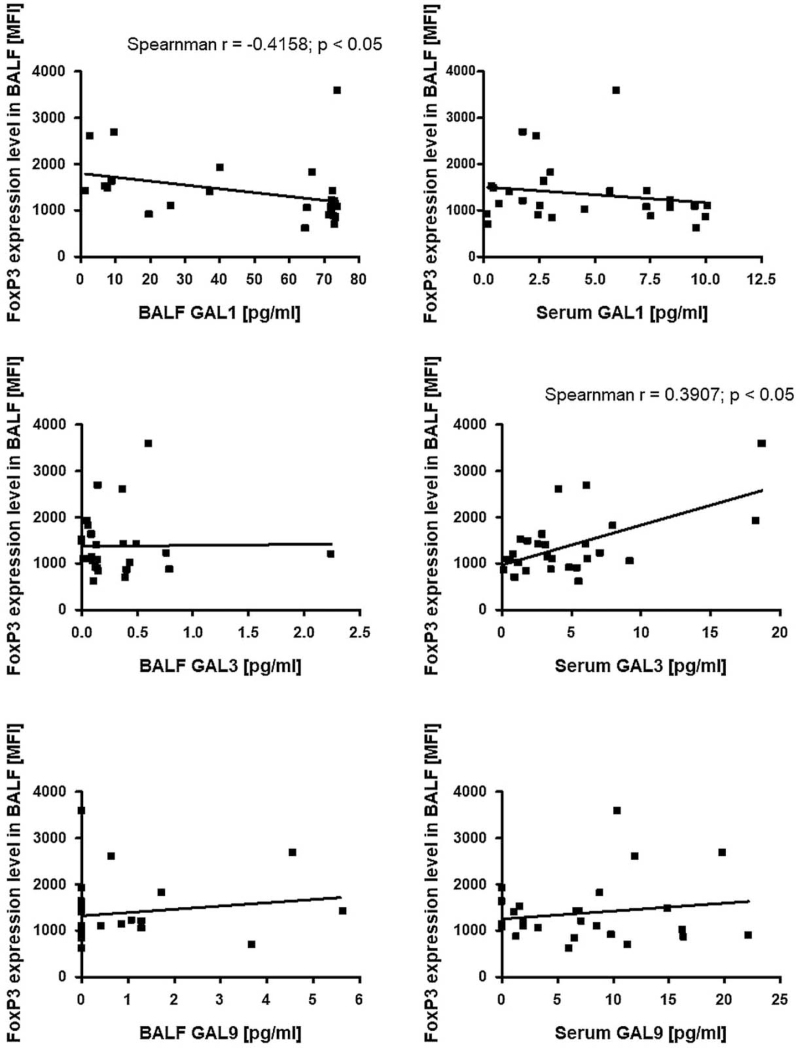
The correlation between FoxP3 expression level in BALF and Gal-1, Gal-3 and Gal-9 in BALF and serum. BALF = bronchoalveolar lavage fluid, FoxP3 = forkhead box P3, Gal-1 = galectin-1, Gal-3 = galectin-3, Gal-9 = galectin-9.

The percentage of Tregs among Th cells in BALF revealed a significant positive correlation with serum Gal-1/TP concentration (r = 0.5482; *P* < .01) and a positive trend with Gal-1/TP concentration in BALF (Fig. 2). A significant negative correlation was observed between the percentage of Tregs among Th cells in BALF and serum Gal-9/TP concentration (r = −0.4656; *P* < .05) and a negative trend with Gal-9/TP concentration in BALF (Fig. 2). Moreover, FoxP3 expression levels in BALF were negatively correlated with Gal-1 concentration in BALF (r = −0.4158; *P* < .05) and presented a negative trend with serum Gal-1 concentration. FoxP3 expression levels in BALF revealed a significant positive correlation with Gal-3 concentration in the serum (r = 0.3907; *P* < .05) and a negative trend in BALF. The trends were only observed between FoxP3 expression levels in BALF: positive with Gal-9 concentration in BALF and negative with Gal-9 concentration in serum (Fig. 5).

The percentage of Tregs in blood Th cells was negatively correlated with BALF Gal-9/TP concentration (r = −0.6054; *P* < .001) and tended to be positively correlated with serum Gal-9/TP concentration (Fig. 3). No association was found between other DAMPs and Tregs frequency in BALF and blood.

**Figure 6:**
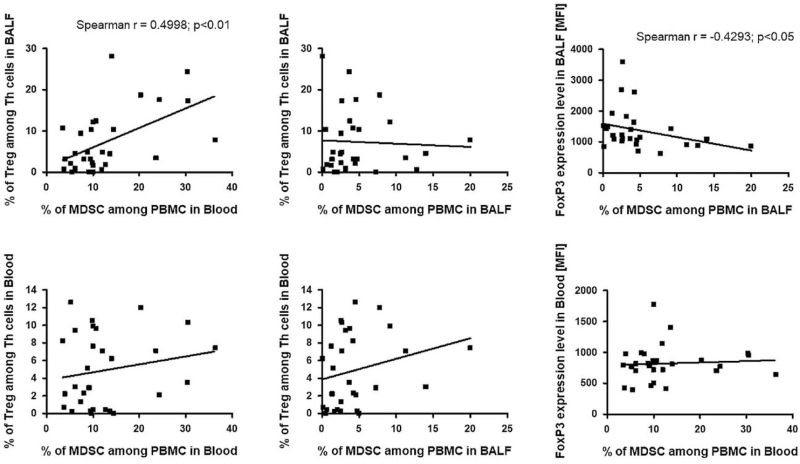
The correlation between Tregs and FoxP3 expression level and MDSCs. FoxP3 = forkhead box P3, MDSC = myeloid-derived suppressor cell, Tregs = regulatory T cells.

The statistical analysis demonstrated a significant positive correlation between the percentage of Tregs among Th cells in BALF and the percentage of MDSCs among peripheral blood mononuclear cell (PBMCs) in the blood (r = 0.4998; *P* < .01) and a positive trend with the percentage of MDSCs among PBMCs in BALF. The rate of Tregs among Th cells in the blood revealed a positive trend with the percentage of MDSCs among PBMCs in the blood and BALF (Fig. 6).

The FoxP3 expression level in BALF was negatively correlated with the percentage of MDSCs among PBMCs in BALF (r = 0.4293; *P* < .05) and showed a positive trend with MDSC among PBMCs in the blood (Fig. 6).

### 3.6 Association between the frequency of Tregs and clinical parameters

An association between the percentage of Tregs, the percentage of Tregs among Th cells, FoxP3 expression level (MFI), and clinical features were evaluated.

**Figure 7:**
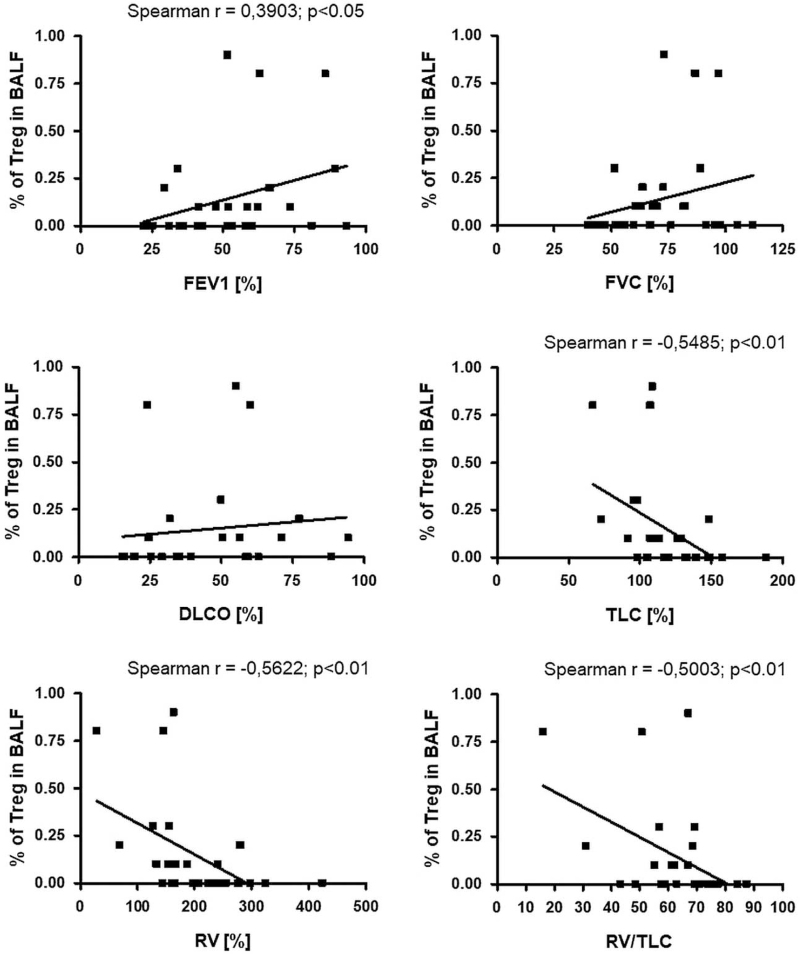
The correlation between Tregs in BALF and pulmonary function tests. BALF = bronchoalveolar lavage fluid, Tregs = regulatory T cells.

The percentage of Tregs in BALF positively correlated with FEV1%pred (r = 0.3903; *P* < .05) and presented a positive trend with FVC%pred and DLCO%pred. A significant negative correlation was observed between the percentage of Tregs and residual volume (RV)%pred (r = −5622; *P* < .01), total lung capacity (TLC)%pred (r = −0.5485; *P* < .01), and RV/TLC (r = −0.5003; *P* < .01). No significant association was found between the percentage of Tregs in the BALF and other clinical parameters (Fig. 7).

**Figure 8:**
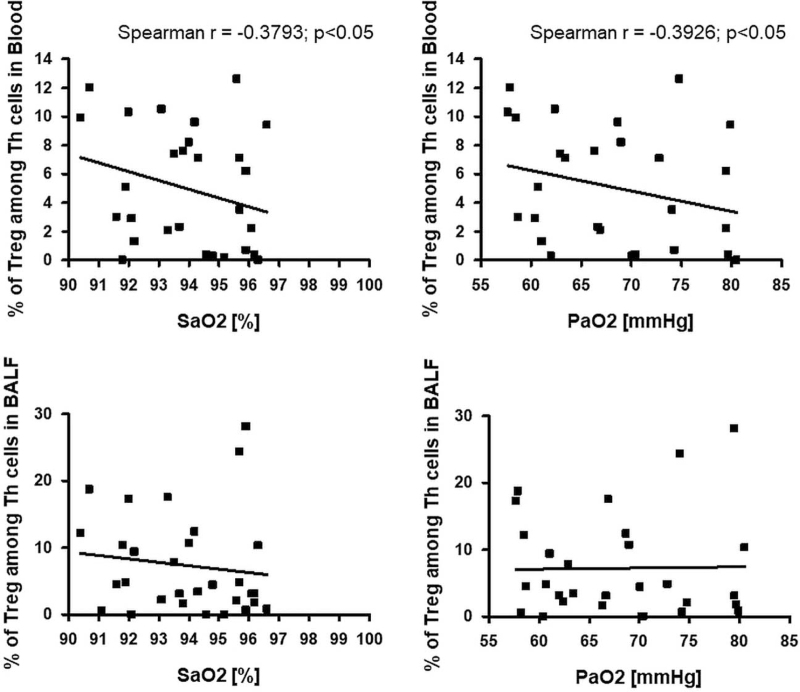
The correlation between Tregs in blood and arterial blood gases parameters. Tregs = regulatory T cells.

Assessing the relationship between the percentage of Tregs among Th cells, we found only a significant negative correlation with the arterial oxygen saturation (r = −0.3793; *P* < .05) and arterial oxygen pressure (PaO_2_) (r = −0.3926; *P* < .05) in blood and a negative trend with these parameters in BALF (Fig. 8).

**Figure 9:**
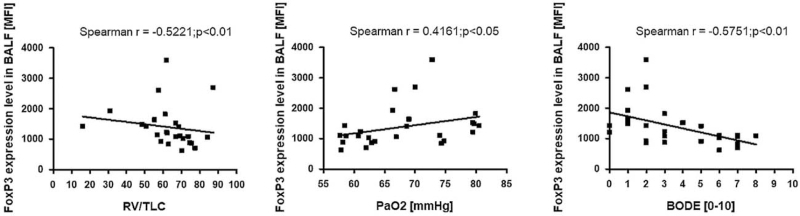
The correlation between FoxP3 expression level in BALF and clinical parameters. BALF = bronchoalveolar lavage fluid, FoxP3 = forkhead box P3.

For FoxP3 expression level, significant correlations were observed only in BALF, which was negatively associated with RV/TLC (r = −0.5221; *P* < .01) and positively correlated with PaO_2_ (r = 0.3926; *P* < .05). Likewise, the FoxP3 expression level was negatively correlated with the BODE index (r = −0.5751; *P* < .01) (Fig. 9).

## 4 Discussion

Our study was driven by the question of whether there are differences in the number of Tregs and FoxP3 in the airway microenvironment and whether there is an association with selected DAMPs and MDSCs that activate an innate and adaptive immune response in the course of COPD.

In the current study, differences between the percentages of Tregs and FoxP3 expression have been shown using BALF and PB. Additionally, our study revealed an association between Tregs, Gal-1, Gal-3, Gal-9, HSP-27, and MDSCs, confirming the link between adaptive and innate immune responses in COPD. The analyzed immunological parameters seem to have valuable clinical implications in the course of COPD.

One of the factors influencing COPD development is the modification of the normal inflammatory response of the respiratory tract to chronic irritants, such as cigarette smoke, and may exist after smoking cessation.^[1]^ This hypothesis suggests that COPD may be an auto-inflammatory disease, and the pathogenic process may be associated with uncontrolled local and systemic immune responses.^[33]^ Previous studies^[8,34–36]^ have shown that T cells play a pivotal role in regulating airway inflammatory processes in COPD. In this group of cells, Tregs are critical for maintaining immune tolerance by suppressing or downregulating the induction and proliferation of effector T cells.^[34]^ Tregs consist of several forms, among which the best researched are those that are CD4^+^, CD25^+^, and FoxP3^+^ as opposed to Th17 cells.^[34,35]^ The transcription factor Foxp3 seems to be a crucial marker because its presence and upregulated expression are required for Tregs development and function, preventing autoimmune diseases.^[30]^ Previous studies have reported the occurrence of Tregs in various COPD materials. Smyth et al^[36]^ reported that chronic cigarette smoke exposure increased airway Tregs numbers in CD4 CD25 bright cells.

In contrast, Lee et al^[37]^ and Barceló et al^[38]^ reported decreased Tregs in patients with emphysema and COPD compared to healthy subjects. Our study showed different percentages of Tregs in the BALF and PB. Tregs accounted for a higher percentage of Th cells in the PB as compared to BALF. Moreover, previously dynamic changes in the blood FoxP3 expression in different COPD phases have been observed.^[39]^ It increases during exacerbation when the inflammatory process aggravates.^[39]^ We found significantly increased FoxP3 expression in Tregs from BALF as compared to that in the PB. Our COPD patient group was in a clinically stable phase of the disease, where the main inflammatory process occurs in the lung microenvironment, and Tregs FoxP3 are involved in controlling pulmonary inflammation by stopping immune responses.^[34]^ This phenomenon may explain our observation. The increased FoxP3 expression in Tregs from BALF, and on the other hand lower percentage of Tregs in blood, may be associated with the slower displacement of mature cells into the lungs where the inflammatory process persists. In the current study, higher expression of FoxP3 in Tregs presented in the lung microenvironment may be related with soluble regulatory factors as cytokines which enhance expression of this transcription factor, and which are abundant in this microenvironmemt.

Previously published data by our team^[12,29,31]^ revealed that MDSCs and DAMPs may be involved in local inflammation in COPD and may intensify the inflammatory process concerning the severity of bronchoconstriction. In the current study, we attempted to determine the relationship between clinical parameters and Tregs in the lower airways, where the inflammatory process begins and persists, and in the PB, as a sign of systemic inflammation. MDSCs are a varied population of cells at different maturation stages produced in the bone marrow.^[40]^ In the microenvironment, these cells mainly suppress T cell function. They not only inhibit the proliferation of T lymphocytes but also induce the accumulation of suppressive regulatory T lymphocytes (Treg FoxP3^+^).^[41]^ This theory was confirmed in the current study, revealing a positive relationship between the percentage of Tregs among Th cells and MDSCs in BALF and blood. MDSCs induce Treg expansion via secretion of IL-10 and TGF-β. In some cancer models, induction of Tregs by MDSCs is associated with cytotoxic lymphocyte antigen 4 expression.^[42]^ Contrary to this expectation, this study found that FoxP3 expression levels were negatively related to MDSCs in BALF. These data may be related to local inflammation intensity and require further research.

DAMPs are endogenous particles secreted by both viable and dying cells. It works as an alert to the organism against the negative consequences of tissue damage and initiates the tissue repair process, including the lung microenvironment.^[43–45]^ One group of DAMPs is heat shock proteins (HSPs), which are implicated in innate and adaptive immune systems. They can stimulate dendritic cells, natural killer cells, and macrophages.^[46]^ Both autologous and recombinant HSPs stimulate T-cell proliferation and immunomodulatory functions.^[47,48]^ They are necessary to induce T-cell phenotypes and are essential in the induction, proliferation, suppressive function, and cytokine production of Tregs. HSPs induce Tregs that can suppress autoimmunity.^[46]^ The current study supports this observation in the COPD group. The HSP27 concentration was positively correlated with the percentage of Tregs in both BALF and blood. We also assessed the relationship between Gal-1, Gal-3, Gal-9, and Tregs in the present study. Gal-1 and Gal-3 are known to have the strongest pro-inflammatory profile and may contribute to the innate immune response involved in the pathogenesis of COPD.^[49]^ The current study presented a significant positive correlation between the percentage of Tregs among Th cells in BALF with Gal-1/TP concentrations in serum and a positive trend with the concentration of Gal-1/TP in BALF. Cibrián and Sánchez-madrid^[50]^ supported our results suggesting that CD69 ligands, such as Gal-1, may induce signaling via regulation not only during cognate contacts between T cells and antigen-presenting cells in lymphoid organs but also in the periphery, where cytokines and other metabolites control the outcome of the immune response. Gal-9 is a multifunctional protein that participates in several cellular processes. However, the mechanism by which Gal-9 and its isoforms/splice variants are regulated at the expression, polyadenylation, posttranslational modification, and secretory levels is by far unclear.^[51]^ A recent study^[52]^ showed that Gal-9 downregulates Th1 and Th17 cell responses and is related to suppression mediated by Tregs in murine autoimmune disease models. Horio et al^[52]^ assessed BALF Gal-9 in a murine model and indicated that it may protect against PPE-induced inflammation and emphysema. Contrary to our expectations, this study revealed a negative correlation between the percentage of Tregs among Th cells in BALF and serum Gal-9/TP concentration and a negative correlation with Gal-9/TP concentration in BALF. Moreover, FoxP3 expression level in BALF was negatively correlated with Gal-1 concentration in BALF and presented a negative trend with serum Gal-1 concentration, which may be related to the greater severity of local inflammation and recruitment of Tregs into the lung microenvironment.

To the best of our knowledge, only a few studies have assessed the connection between Tregs, HSP-27, galectins, and MDSCs in BALF from COPD patients. The results of the current study provide new information about the possible relationship between them in the course of COPD. Additionally, we attempted to reflect the immunological markers assessed on clinical parameters characterizing patients with COPD. Tregs are known to stop immune responses after invading pathogens or noxious particles and may promote tissue repair and regeneration.^[34]^ Hou et al^[8]^ showed that Tregs in BALF were positively correlated with COPD severity, as assessed using FEV1%pred. Farahani et al^[9]^ reported similar results. They found a positive correlation between IL-10, a cytokine related to Tregs, and FEV1%pred in the COPD group. Similar results were obtained by Zheng et al,^[53]^ by using lung tissue and blood. In this study, Treg% positively correlated with FEV1%, FEVC%, FEV1/FEVC,^[53]^ which confirmed our results. We also found a positive relationship between BALF Tregs and FEV1%pred and FVC% pred. Based on previous studies^[54–57]^ conducted on mouse and rat models, a decrease in the percentage of Tregs promotes the development of emphysema. Our study results correspond to those mentioned above. Tregs present in BALFs were negatively correlated with RV%pred, TLC%pred, and BALF FoxP3 expression with the RV/TLC ratio. Additionally, we found positive relationships between Tregs in BALF and DLCO%pred. The estimated pulmonary function parameters were closely associated with the clinical diagnosis of emphysema in patients with COPD.

Tissue hypoxia occurs following tissue injury, infection, inflammation, and rapid tumor growth. Residents or recruited immune cells need to adapt rapidly to a hypoxic environment.^[58]^ Hypoxia in solid tumors promotes the secretion of chemokines and activation of their receptors. This may promote the mobilization of various cell types in the body and the recruitment of Tregs to tumor sites.^[59]^ The current study results align with these observations because we found negative relationships between peripheral % of Tregs among Th cells and arterial oxygen saturation and PaO_2_. However, contrary to our expectations, we observed that the lung microenvironment FoxP3 expression was positively related to PaO_2_. This may be related to the locally intensified inflammatory process.

Interestingly, we observed a positive correlation between FoxP3 expression in BALF and the BODE index, which integrates BMI, airflow limitation (FEV1), dyspnea, and 6-minute walk distance, predicts mortality in COPD.^[18]^ This study is the first to present such a link. This suggests that Tregs FoxP3 expression in the lung microenvironment may be indirectly associated with COPD prognosis.

Our study has several limitations. The first is the lack of a control group. It was impossible to obtain consent from the ethics committee to perform VBF in healthy subjects. Second, we did not investigate 3 distinct subpopulations of Tregs: CD25^++^ CD45RA^+^ resting Tregs, CD25^+++^ CD45RA^−^ activated Tregs, which are suppressive, and CD25^++^ CD45RA^−^ cytokine-secreting (Fr III) cells with pro-inflammatory capacity.^[8]^ In this study, the entire Tregs population was assessed. Third, it is difficult to define the immunophenotype of MDSCs in humans. These cells are positive for myeloid lineage markers, CD11b, and CD33, but do not express HLA-DR molecules, and are negative for markers characteristic of lymphoid cells. Recently, MDSCs were subdivided into monocytic (CD14^+^/HLA^−^DR^−^) and granulocytic (CD15^+^/HLA^−^DR^−^) MDSCs. In the present study, MDSCs were defined as cells belonging to leukocytes (CD45^+^) with low granularity (SSClow), and expressed the following immunophenotype: CD11b^+^/CD33^+^/HLA^−^DR^−^.^[31,60]^

## 5 Conclusions

In conclusion, our study demonstrated an interaction between innate and adaptive immune responses in COPD patients, especially on Tregs, MDSCs, and DAMPs in the airway microenvironment, using BALF. This study presented the conversion of the immune response to clinical parameters that may be associated with disease severity. Additionally, the expression of Tregs in BALF may have prognostic potential in patients with COPD.

## Acknowledgments

The authors would like to thank the participants for agreeing to participate in the study and Tomasz Trafas (PhD) for statistical analysis.

## Author contributions

Conceptualization, HB-G, BB-L, AN, MK, JS; methodology, BB-L, MK, and JS; software, BB-L and AN; validation, HB-G; formal analysis, BB-L, MK, investigation, BB-L, TP, AN, MK, MW, and SY; resources, HB-G; data curation, BB-L, MK; writing-original draft preparation, BB-L, MK; writing-review and editing, BB-L, MK, MS-M, and HB-G; visualization, BB-L; supervision, HB-G; project administration, BB-L; funding acquisition, HB-G. All authors have read and agreed to the published version of the manuscript.

**Conceptualization:** Agata Nowicka, Beata Brajer-Luftmann, Halina Batura-Gabryel, Jan Sikora, Mariusz Kaczmarek.

**Data curation:** Beata Brajer-Luftmann, Mariusz Kaczmarek.

**Formal analysis:** Beata Brajer-Luftmann, Mariusz Kaczmarek.

**Funding acquisition:** Halina Batura-Gabryel.

**Investigation:** Agata Nowicka, Beata Brajer-Luftmann, Magdalena Wyrzykiewicz, Mariusz Kaczmarek, Senan Yasar, Tomasz Piorunek.

**Methodology:** Beata Brajer-Luftmann, Mariusz Kaczmarek.

**Project administration:** Beata Brajer-Luftmann.

**Resources:** Halina Batura-Gabryel.

**Software:** Agata Nowicka, Beata Brajer-Luftmann.

**Supervision:** Halina Batura-Gabryel.

**Validation:** Halina Batura-Gabryel.

**Visualization:** Beata Brajer-Luftmann.

**Writing – original draft:** Beata Brajer-Luftmann, Mariusz Kaczmarek.

**Writing – review & editing:** Beata Brajer-Luftmann: Halina Batura-Gabryel, Mariusz Kaczmarek, Marta Stelmach-Mardas.
